# Supplementary material for: Terahertz pulse shaping using diffractive surfaces
Source: Nat Commun. 2021 Jan 4;12:37. doi: 10.1038/s41467-020-20268-z (PMC7782497; doi:10.1038/s41467-020-20268-z)
Supplement: Supplementary file 1 — Supplementary Information [file 41467_2020_20268_MOESM1_ESM.pdf]

Supplementary Information

Terahertz Pulse Shaping Using Diffractive Surfaces

Muhammed Veli[a,b,c], Deniz Mengu[a,b,c], Nezih T. Yardimci[a,b,c], Yi Luo[a,b,c], Jingxi Li[a,b,c], Yair Rivenson[a,b,c], Mona Jarrahi[a,c], Aydogan Ozcan*[abc]

[a]*Department of Electrical & Computer Engineering, University of California Los Angeles (UCLA), California, USA*

[b]*Department of Bioengineering, University of California Los Angeles (UCLA), California, USA*

[c]*California NanoSystems Institute (CNSI), University of California Los Angeles (UCLA), California, USA*

**E-mail: ozcan@ucla.edu*

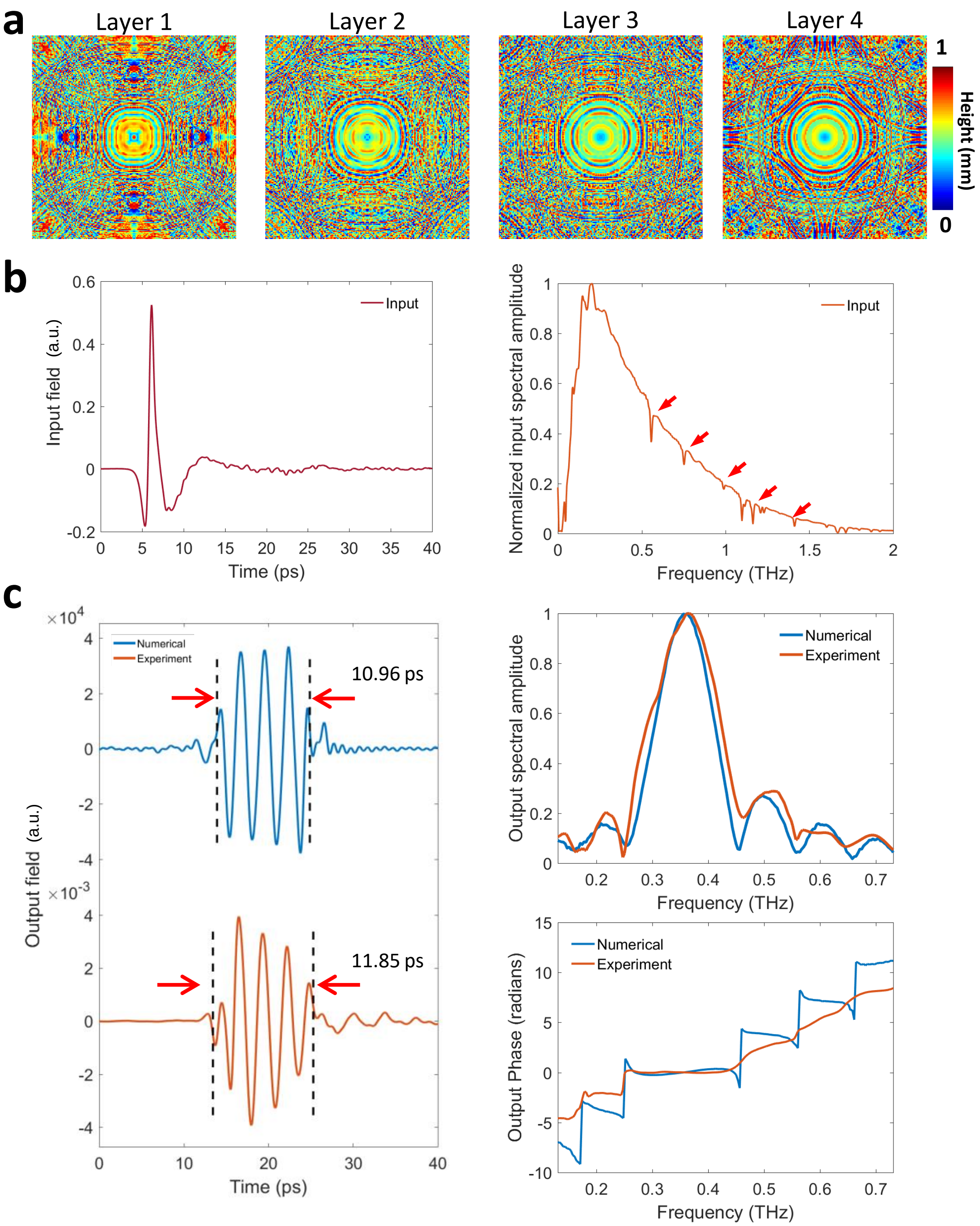

Supplementary Figure 1 Pulse shaping diffractive network design and output results a The thickness profiles of the resulting diffractive layers after deep learning-based training in a computer. These diffractive layers synthesize a square pulse with a width of 10.96 ps over the output aperture for an input pulse shown in **b**. **b** Normalized amplitude of the input terahertz pulse measured right after the input aperture (see Fig. 1 of the main text); in time-domain (left) and spectral domain (right). The red arrows on the measured spectral amplitude profile represent the water absorption bands at terahertz frequencies. **c Left**: The numerically computed (blue) and the experimentally measured (orange) output pulses in time domain. **Top right**: The normalized spectral amplitudes corresponding to the numerically computed (blue) and the experimentally measured (orange) pulses. **Bottom right**: Unwrapped spectral phase distributions computed based on the numerical forward model (blue) and the experimentally measured (orange) pulse.

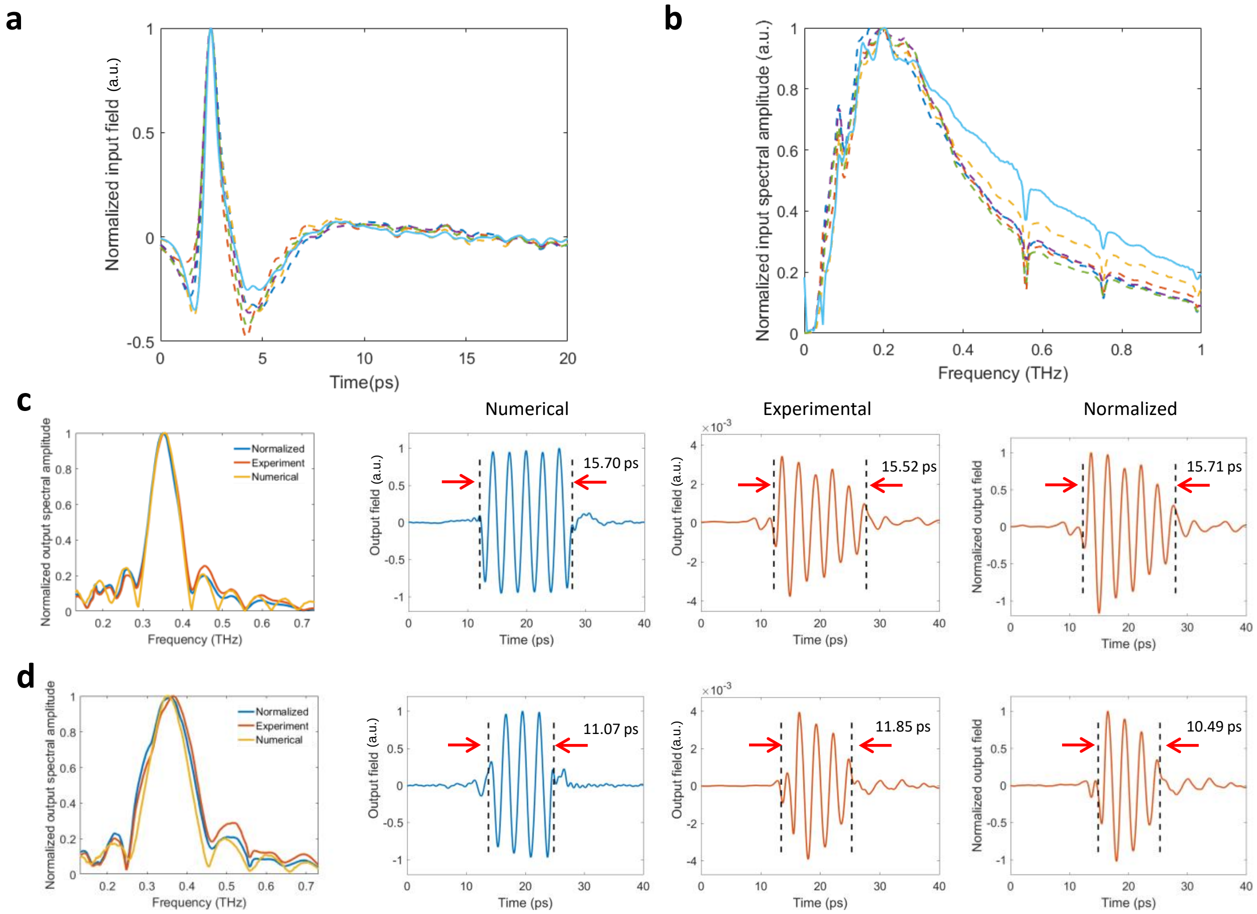

Supplementary Figure 2 Spectral normalization of the output pulse. a Input terahertz pulses impinging upon the diffractive network. Dashed lines represent the input pulses that were used in the training phase and the solid line represents the actual experimental input pulse used in the testing phase. **b** Normalized spectral amplitudes of the input terahertz pulses shown in **a**. **c, d** Left: normalized spectral amplitude of the output pulse obtained at the end of the training phase (yellow), compared with the experimental spectral amplitude before (orange) and after the spectral normalization step (blue). Right: numerical training output field (blue), experimental output field (orange) and the normalized output field (orange) corresponding to the desired (ground truth) square pulses with pulse-widths of **(c)** 15.49 ps and **(d)** 10.52 ps.

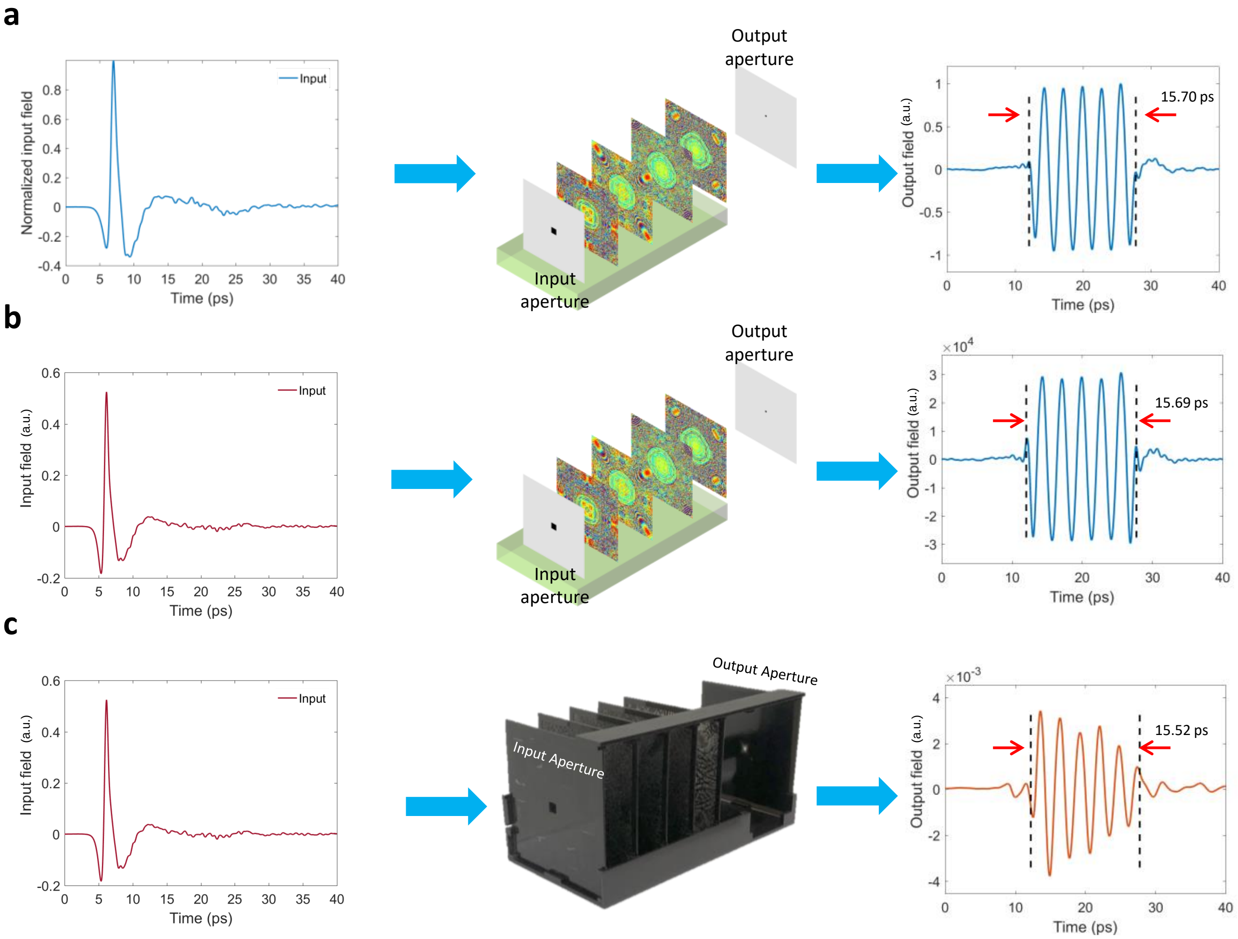

Supplementary Figure 3 The temporal profiles of the output pulses resulting from the designed numerical model (a, b) and the 3D-fabricated diffractive network (c) that was trained to synthesize a 15.7 ps square pulse. (a) the input pulse is one of the pulses that were used in training phase; (b,c) the input pulse is experimentally measured at the input aperture.

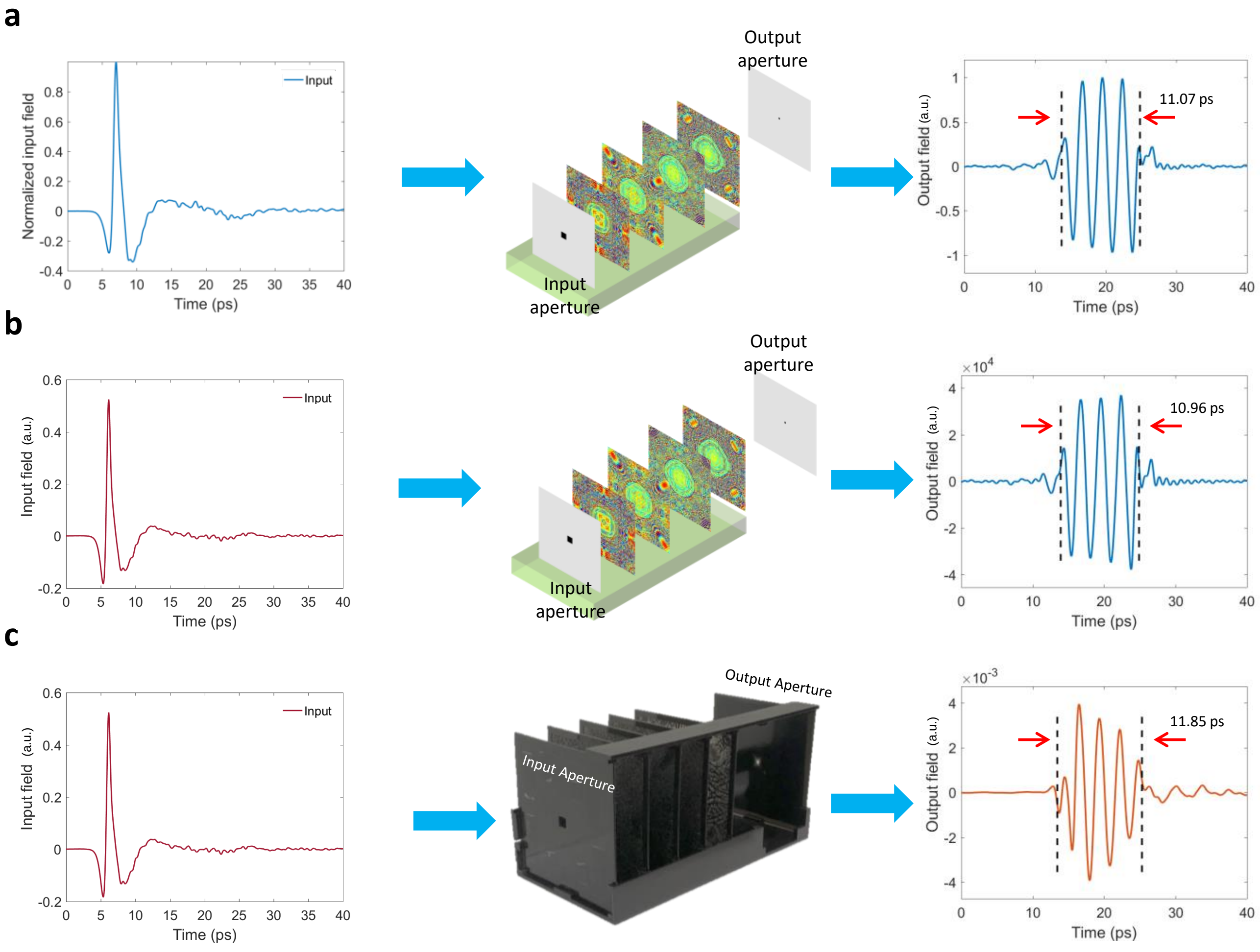

Supplementary Figure 4 The temporal profiles of the output pulses resulting from the designed numerical model (a, b) and the 3D-fabricated diffractive network (c) that was trained to synthesize a 11.07 ps square pulse. (a) the input pulse is one of the pulses that were used in training phase; (b,c) the input pulse is experimentally measured at the input aperture.

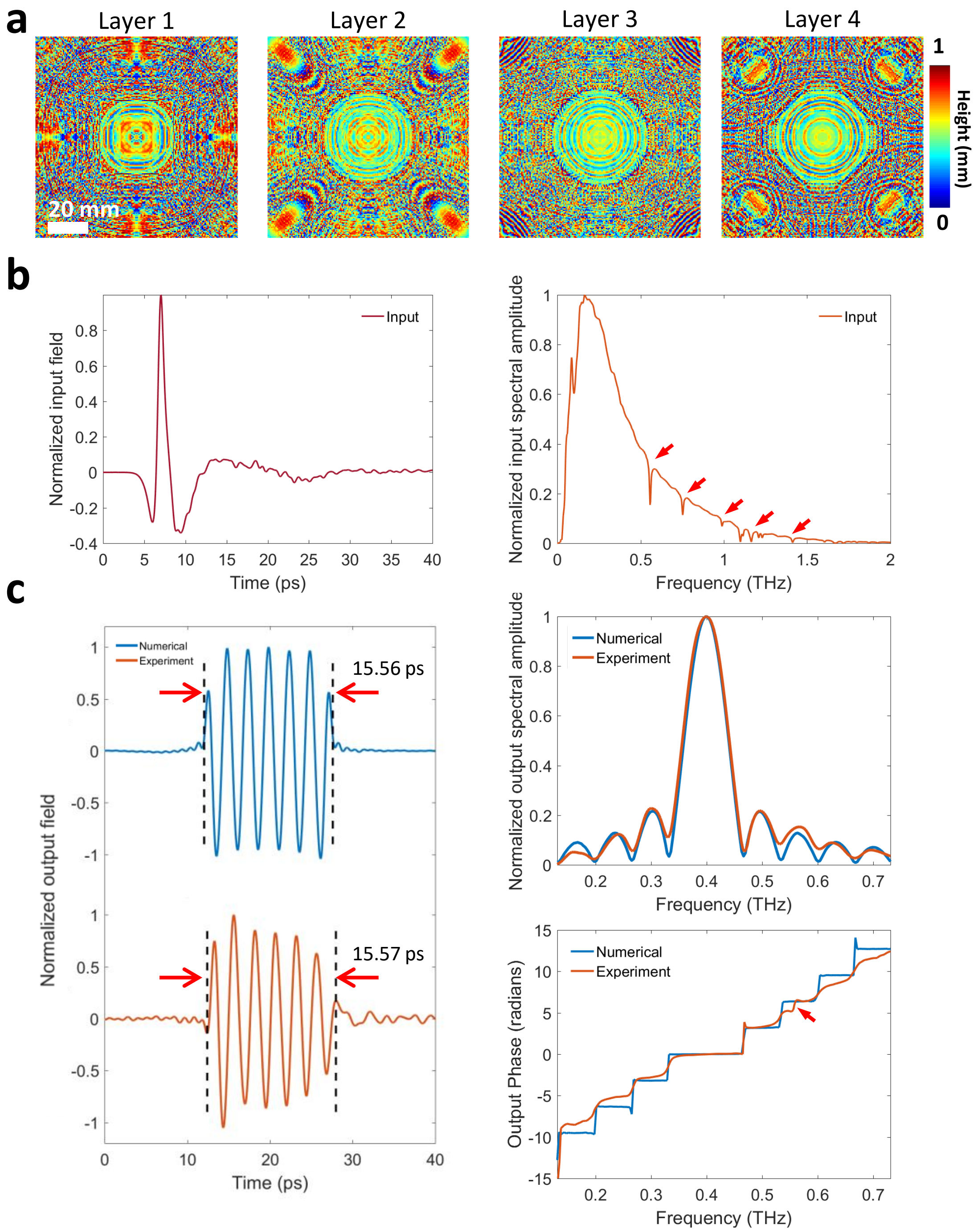

Supplementary Figure 5 Generic pulse shaping diffractive network design and output results. a The thickness profiles of the resulting diffractive layers after deep learning-based training in a computer. These diffractive layers synthesize a square pulse with a width of 15.56 ps over the output aperture for an input pulse shown in **b**. **b** Normalized amplitude of the input terahertz pulse measured right after the input aperture (see Fig. 1 of main text); in time-domain (left) and spectral domain (right). The red arrows on the measured spectral amplitude profile represent the water absorption bands at terahertz frequencies. **c Left:** The numerically computed (blue) and the experimentally measured (orange) output pulses in time domain. **Top right:** The normalized spectral amplitudes corresponding to the numerically computed (blue) and the experimentally measured (orange) pulses. **Bottom right:** Unwrapped spectral phase distributions computed based on the numerical forward model (blue) and experimentally measured (orange) pulse. Red arrow on the phase profile exemplifies a small discrepancy between the numerical and the experimental results due to the water absorption bands at THz frequencies.

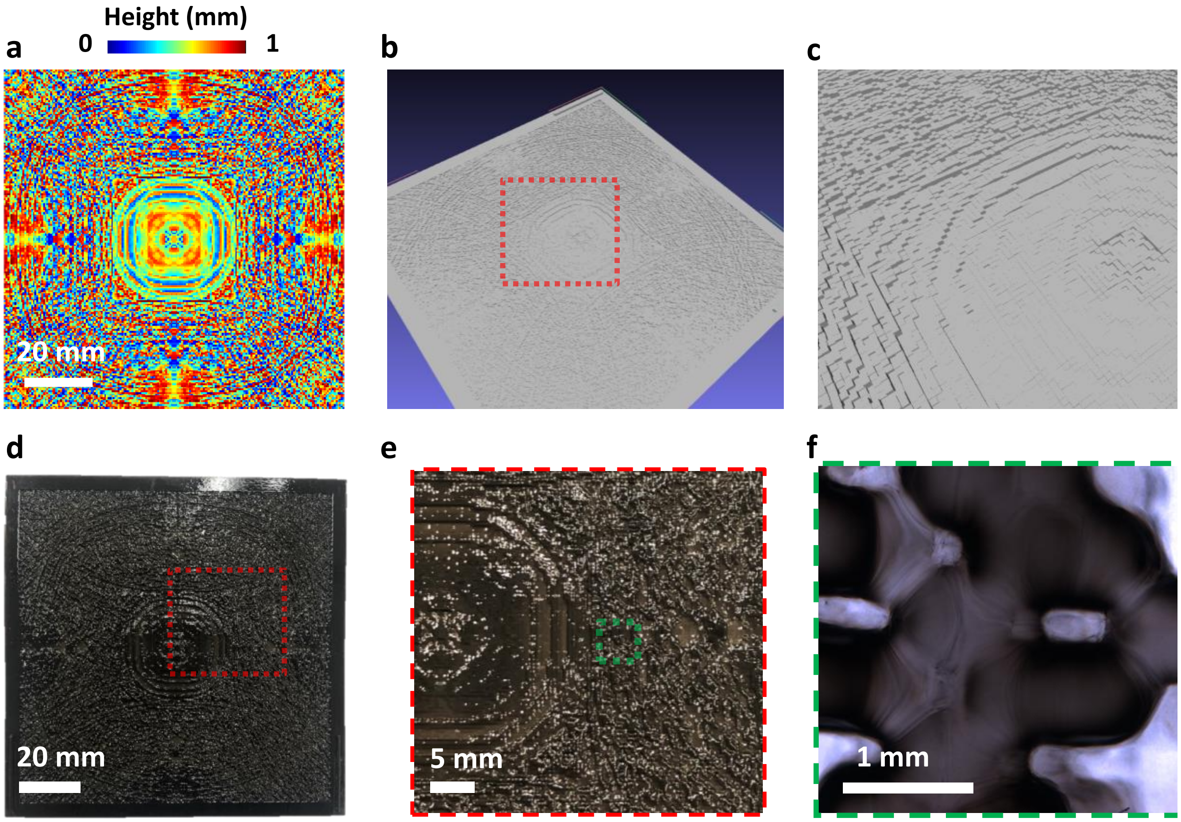

Supplementary Figure 6 (a) The thickness profile of the first layer of the diffractive network reported in Supplementary Fig. 5. **(b)** CAD drawing of the same layer and **(c)** zoomed-in version of the red square shown in (b). **(d)** The photo of the layer presented in (a), **(e)** zoomed-in version of the red square shown in (d) and **(f)** a microscopic image showing the fabricated structural details of the green square in (e).

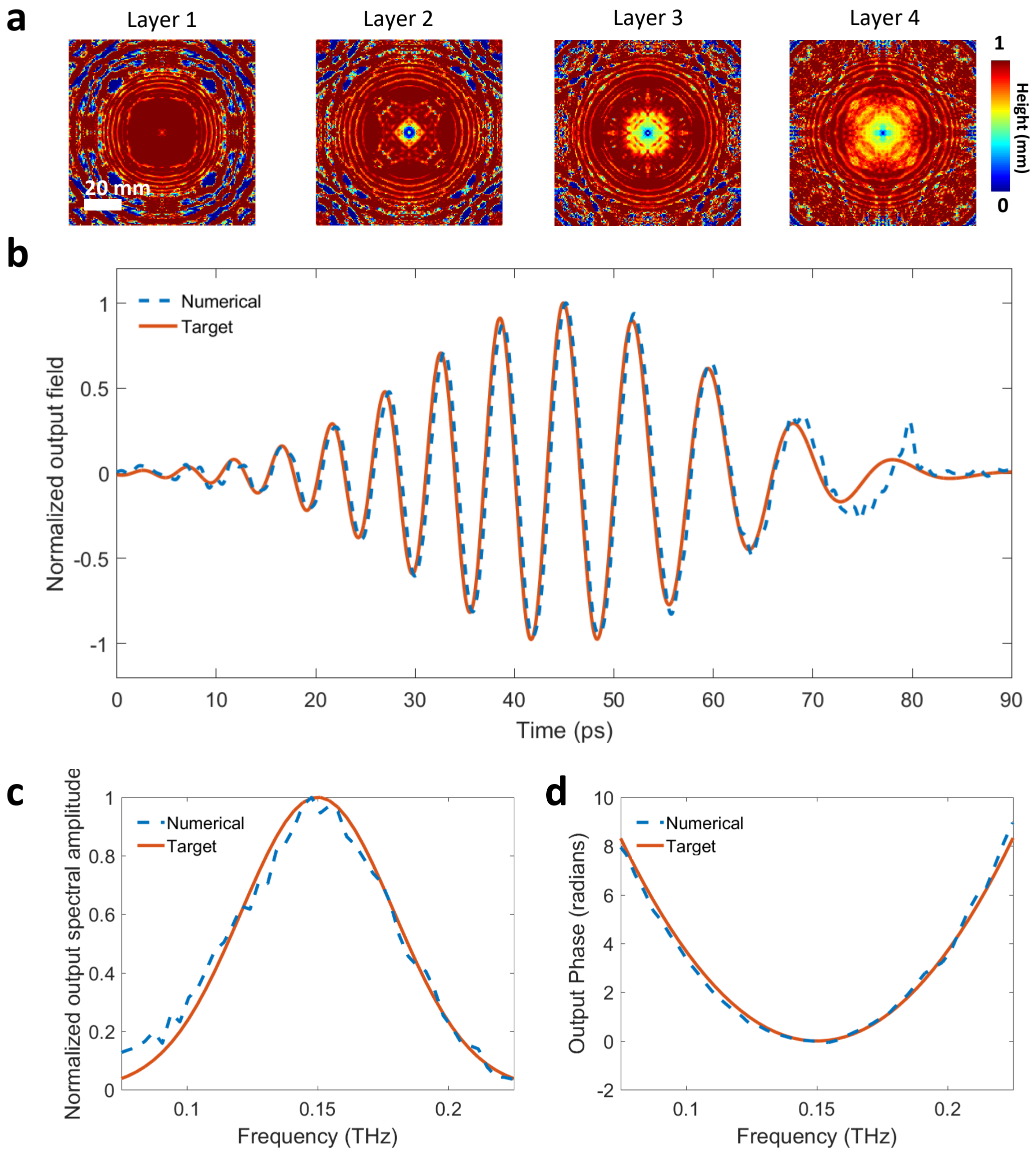

Supplementary Figure 7 Generic pulse shaping diffractive network design trained for synthesizing a chirped Gaussian pulse. a The thickness profiles of the resulting diffractive layers after deep learning-based training in a computer. These diffractive layers synthesize a chirped Gaussian pulse over the output aperture of the diffractive network. **b** The numerically computed (dashed blue) and the targeted ground-truth (orange) output pulses in time domain. **c** The normalized spectral amplitudes corresponding to the numerically computed (dashed blue) and the target (orange) pulses. **d** Unwrapped spectral phase distributions computed based on the numerical forward model (dashed blue) and the target (orange) pulse.

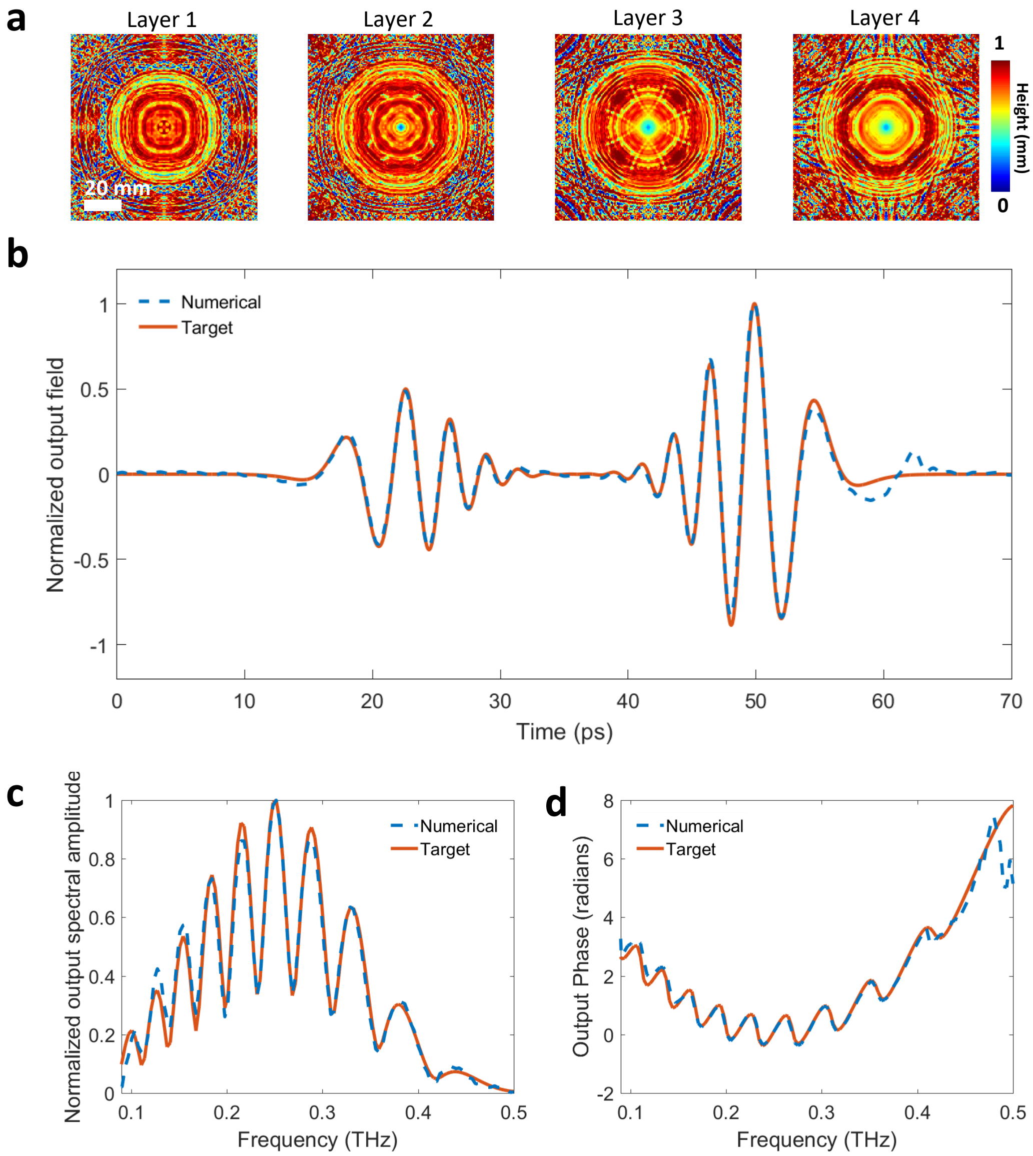

Supplementary Figure 8 Generic pulse shaping diffractive network design trained for synthesizing a sequence of positive and negative chirped Gaussian pulses. a The thickness profiles of the resulting diffractive layers after deep learning-based training in a computer. These diffractive layers synthesize a sequence of positive and negative chirped Gaussian pulses over the output aperture of the diffractive network. **b** The numerically computed (dashed blue) and the targeted ground-truth (orange) output pulses in time domain. **c** The normalized spectral amplitudes corresponding to the numerically computed (dashed blue) and the target (orange) pulses. **d** Unwrapped spectral phase distributions computed based on the numerical forward model (dashed blue) and target (orange) pulse.

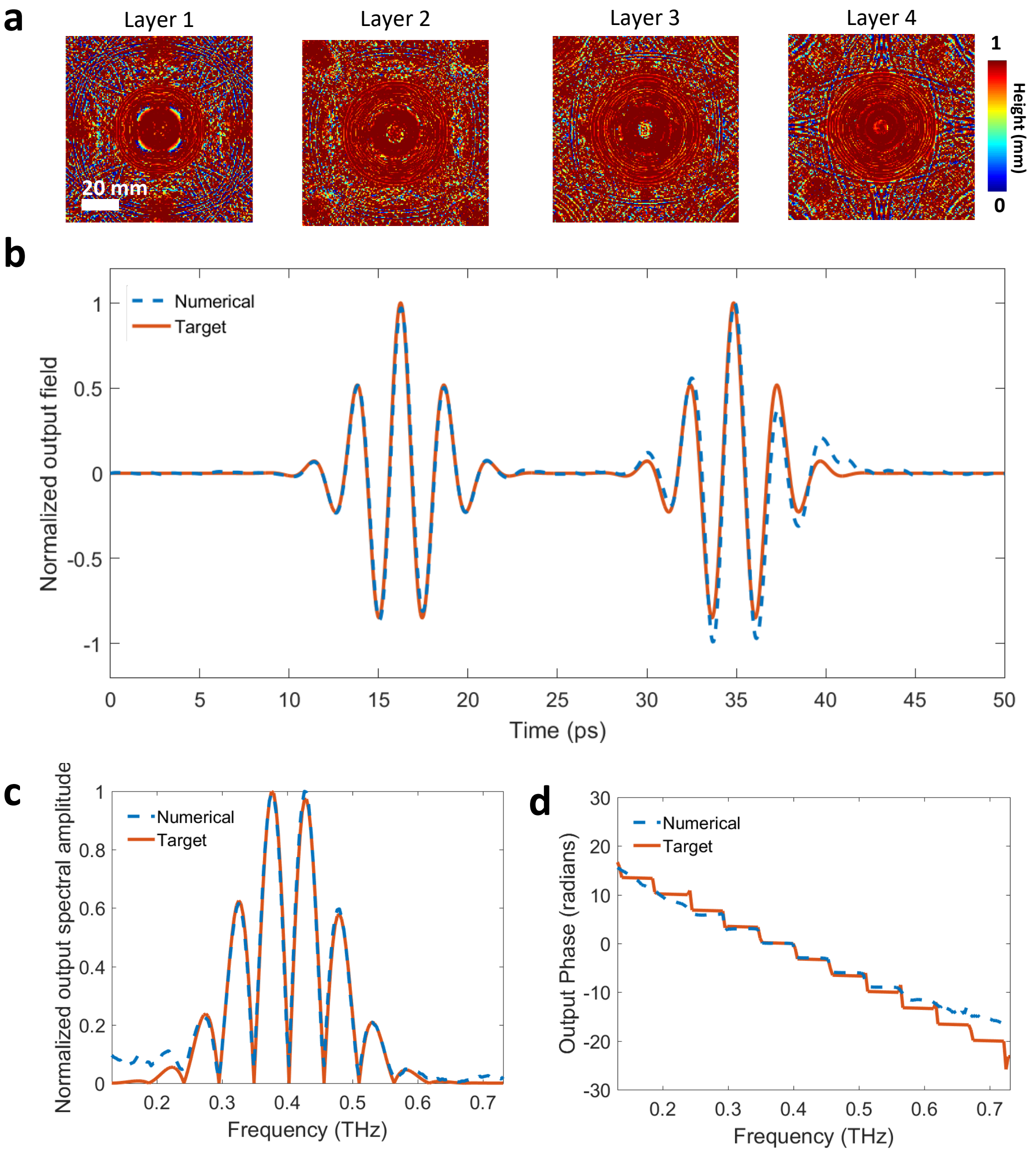

Supplementary Figure 9 Generic pulse shaping diffractive network design trained for synthesizing a sequence of chirp-free Gaussian pulses. a The thickness profiles of the resulting diffractive layers after deep learning-based training in a computer. These diffractive layers synthesize a sequence of chirp-free Gaussian pulses over the output aperture of the diffractive network**. b** The numerically computed (dashed blue) and targeted ground-truth (orange) output pulses in time domain. **c** The normalized spectral amplitudes corresponding to the numerically computed (dashed blue) and the target (orange) pulses. **d** Unwrapped spectral phase distributions computed based on the numerical forward model (dashed blue) and the target (orange) pulse.

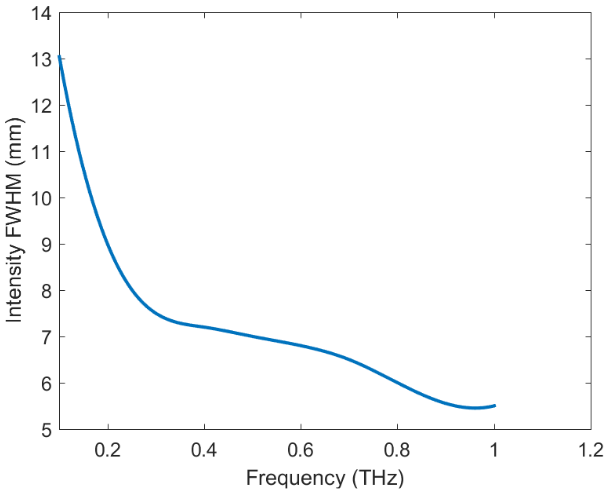

Supplementary Figure 10 Experimentally measured full width at half maximum (FWHM) values of the spatial intensity profiles of different spectral components in the THz beam at the input aperture plane of the diffractive network.
